# A "London" Certificated Nurse on "London" Nurses and Their Treatment

**Published:** 1918-09-28

**Authors:** 


*To the Editor of* The Hospital.

### A "London" Certificated Nurse on "London" Nurses and their Treatment.

*To the Editor of* The Hospital.

Sir,—You have already given much of your valuable space to the question of the London Hospital nurses. May I, as one of them, ask for room to reply to the letter written by Dr. Chapple in your last issue. Dr. Chapple's letter gives the public and other nursing institutions a very wrong impression of the conditions provided for a private nurse by the authorities here.

A candidate when applying for admission to the London Hospital training-school is informed in the regulations that at the end of her training she will be appointed to serve on the private staff or hospital staff as required. She is also told in the regulations what salary she will be paid, and, what is more, she has a personal interview with the matron or her assistant before being accepted for training, when she has every opportunity of asking any questions she desires with reference to how the time will be spent at the "London" if she decides to get her training there. What more can a candidate expect? If she is not satisfied with the prevailing conditions at the "London," then is the time for her to make up her mind as to whether she will get her training there or elsewhere.

Supposing she does decide to train at the "London," what are the conditions she actually finds there? If she is ill she is cared for until she is well again; and not only that, her salary is paid all the time until she is fit to return to work, and there is no limit to the time she may be off duty on sick leave.

If there are difficulties about going home or to friends for sick leave, it is the hospital authorities who arrange for her convalescence at the sea or in the country. She is paid during her holiday, she gets a full-pay pension for life at the end of eighteen years at the age of forty-five, and, what is more, she is then free to take her pension, and, if she wishes to, she can increase her income by getting work elsewhere. If any relatives are ill, however inconvenient it must be at times, she is released from duty to attend to them, and frequently the best medical advice is given, with no expense to the nurse, by arrangements being made for the admission of the relative to hospital. I wonder how many nurses owe a debt of gratitude to the hospital, and are grateful for the way their relatives have been taken care of there? The conditions already mentioned relate not only to the private nurses, but to the whole nursing staff. A private staff nurse has few expenses and few anxieties, as far as she herself is concerned. She is given indoor and outdoor uniform, which she can always wear on "days off" and holidays, etc. She has no worry as to what will happen to her if she is taken ill; she knows she will be looked after and cared for until she is fit for duty again, and last, but not least, her salary will be paid all the time. She is given a "day off" every fortnight, and these accumulate while she is at a case; but arrangements are made so that she can remain in the Nurses' Home for them and be taken care of if she has no friend to spend them with.

As to her capabilities, surely the public are the best judge of these. Why do they employ us if we are not trained, as Dr. Chapple suggests in his letter? If the public were not grateful to the London Hospital nurse, or satisfied that she was a fit person to employ, why employ us? And yet they do, and, what is more, appear satisfied with us, as proved by Dr. Chapple's statements. After all, a nurse does not become trained in two years without costing the hospital something for her training, board, and lodging, and is it not right that she should give something in return? When she starts her training, she sees the justice of this, and continues to do so. Dr. Chapple speaks of £5,000 profit per annum. Would any private staff nurse wish to put her contribution to this in her pocket, when she is being paid as well as any nurse is paid in these days? (Nurses are underpaid, I grant you.) Would she take anything and give nothing in return? After all, any so-called profits made (so-called because the money is spent on the nurses) do not nearly cover the cost of the nurses in peace-time, and they are much less likely to nowadays, when even an egg costs 6d. Has Dr. Chapple ever seen the way the nurses are cared for at the London? I think if he had he would have a

**Some London Hospital Correspondents**—(*continued*).

different tale to tell of the £5,000 he calls profit. In spite of all the expense and present difficulties in getting food, we are well fed, and I am sure no one will agree with me more than the private nurse as to the truth of this. Comfortable quarters are provided for us, and I can already see the trouble that is being taken to arrange for our comfort this winter, so that we shall not feel the coal shortage, though coal shortage we know there must be in the hospital, as elsewhere. The cost of running the laundry must have increased considerably, but the nursing staff have not suffered in consequence. I have many friends on the private staff, and I am sure they would agree with all I have said. The private nursing staff are not discontented with their lot, that I am sure of; and, after all, it is they who matter.

(Signed) A LONDON HOSPITAL CERTIFICATED NURSE.
September 24, 1918.

